# Cadaveric bone marrow mesenchymal stem cells: first experience treating a patient with large severe burns

**DOI:** 10.1186/s41038-015-0018-4

**Published:** 2015-09-18

**Authors:** Eduardo Mansilla, Gustavo H. Marín, Mirta Berges, Silvia Scafatti, Jaime Rivas, Andrea Núñez, Martin Menvielle, Roberto Lamonega, Cecilia Gardiner, Hugo Drago, Flavio Sturla, Mercedes Portas, Silvia Bossi, Maria Victoria Castuma, Sandra Peña Luengas, Gustavo Roque, Karina Martire, Jose Maria Tau, Gabriel Orlandi, Adrian Tarditti

**Affiliations:** 1 Tissue Engineering, Regenerative Medicine and Cell Therapies Laboratory, CUCAIBA, Province of Buenos Aires Ministry of Health, Transplantation Program, La Plata, Province of Buenos Aires, Argentina; 2 Burns and Plastic Surgery Department, San Martín Hospital, Province of Buenos Aires Ministry of Health, La Plata, Province of Buenos Aires, Argentina; 3 Burns Hospital, Buenos Aires City, Argentina; 4 Department of Chemistry, Mayaguez Campus, University of Puerto Rico, Mayaguez Puerto Rico, USA

**Keywords:** Burns, Cadaveric, Transplantation, Skin regeneration, Mesenchymal stem cells

## Abstract

**Background:**

In January 2005, Rasulov et al. originally published “First experience in the use of bone marrow mesenchymal stem cells (MSCs) for the treatment of a patient with deep skin burns”. Here, we present the first ever treated patient with cadaveric bone marrow mesenchymal stem cells (CMSCs) in the history of Medicine.

**Methods:**

A young man, who severely burned 60 % of his total body surface with 30 % of full-thickness burns while working with a grass trimmer that exploded, was involved in the study. MSCs were obtained from the bone marrow of a cadaver donor in a routine procurement procedure of CUCAIBA, the Province of Buenos Aires, Argentina, Ministry of Health, Transplantation Agency, cultured, expanded, and applied on the burned surfaces using a fibrin spray after early escharotomy.

**Results:**

So far, our preliminary experience and our early results have been very impressive showing an outstanding safety data as well as some impressive good results in the use of CMSCs.

**Conclusions:**

Based on all this, we think that improvements in the use of stem cells for burns might be possible in the near future and a lot of time as well as many lives could be saved by many other research teams all over the world. CMSCs will probably be a real scientific opportunity in Regenerative Medicine as well as in Transplantation.

## Background

In January 2005, Rasulov et al. originally published “First experience in the use of bone marrow mesenchymal stem cells (MSCs) for the treatment of a patient with deep skin burns”. This was the first treatment in the history of medicine that used allogeneic MSCs in a human being with extensive skin burns [superficial to full-thickness skin burn, total area 40 %, full-thickness burn area 30 %]. “Allogeneic fibroblast-like bone marrow MSCs,” as designated at that time by the authors, were placed onto the surface of a deep and large thermal burn of a female patient. The dynamic of healing confirmed a high tempo of wound regeneration in the presence of active neo-angiogenesis after transplantation of these “fibroblast-like MSCs”. Due to this, auto-dermoplasty of the burn wounds could be carried out with good results as early as on day 4 after transplantation of the stem cells, leading to a more rapid healing of the donor zones and an accelerated rehabilitation of the patient [1]. During that same month of January 2005, we published “Human mesenchymal stem cells are tolerized by mice and improve skin and spinal cord injuries,” in which we presented an accelerated healing process of injured mice skins and spinal cords obtained by the use of xenogeneic (human to mice) MSCs. In this way, immunological barriers were trespassed beyond species without no rejection or adverse reactions to the transplanted human stem cells or the mice, respectively, with very good clinical results [2]. In 2006, we found and described for the first time, “Bloodstream cells phenotypically identical to human mesenchymal bone marrow stem cells circulating in large amounts under the influence of acute large skin damage”. This was without any doubt, a new and paradigmatic evidence for the future use of these cells in regenerative medicine procedures, specially for the treatment of human large burns. We also originally proposed in this paper that it could be possible to mobilize MSCs from the bone marrow by induced therapeutic ways. These pharmacological mechanisms could be very similar to those biochemical alarm signals, naturally produced from damaged tissues, in order to accomplish many regenerative mechanisms [3]. In December 2010, we observed and published an “Outstanding survival and regeneration process by the use of intelligent acellular dermal matrices and MSCs”. This study was done in a burned pig model in which an “intelligent” acellular biological pig dermis with nanoparticles and fibers coated with an anti-CD44 monoclonal antibody and loaded with growth factors like GM-CSF were implanted in a deep severe wound bed after escharotomy of the burned tissue [4]. In 2011, we presented to our Argentine regulatory authority for organ, tissue and cell transplantation (INCUCAI) the first clinical trial protocol ever being designed in the world using cadaveric bone marrow mesenchymal stem cells (CMSCs) for the treatment of large burns. This was communicated in an editorial to Burns: “Time and regeneration in burns treatment: heading into the first worldwide clinical trial with cadaveric mesenchymal stem cells”, in which we made special considerations about the importance of time in the accomplishment of the regeneration processes of wound healing, specially when stem cells are used [5]. As this trial was approved (including the INCUCAI Ethic Committee review process and the signed consent form), we treated the first patient by the end of that year. CMSCs will probably be a real scientific opportunity in Regenerative Medicine as well as in Transplantation [6]. We were the first research group in the world to use them for regenerative purposes [5, 7]. There is still a great potential in these cells to be discovered. They might significantly change in the near future the possible sources for stem cells obtention. CMSCs could be very important tools to treat burns as well as many other diseases [8–14]. By now, we have made just a little advance in this new field of regenerative medicine by using CMSCs for the treatment of large and deep burns. Here, we present the first experience in the world with this kind of cell treatment applied to a severe burned patient after more than 3 years of follow-up.

## Methods

### Obtention and preparation of human CMSCs for transplantation

Bone marrow blood was obtained by needle aspiration from the iliac bone of a unique cadaver donor, in a routine procurement procedure of our Province of Buenos Aires, Argentina, Ministry of Health, Transplantation Agency, CUCAIBA, after cardiac arrest and when all other organs and tissues for transplantation were already collected. In this procurement procedure, the donor was selected by his negative tests for all adventitious agents, including hepatitis B and C, AIDS, syphilis, cytomegalovirus, and chagas, among many others, as needed by law, for organ and tissue transplantation. Briefly, 10 ml of heparinized bone marrow blood was sterile and transported to our Tissue Engineering, Regenerative Medicine, and Cell Therapies Laboratory in La Plata, Argentina, and processed in a clean room under sterile and GMP conditions. Each 2 ml of this bone marrow blood was diluted in 15 ml of low-glucose DMEM (Gibco/Life Technologies) and cultured for the isolation of MSC in 75 cm^2^ tissue culture flasks at 37 °C in a 5 % CO_2_ incubator at 95 % humidity with 10 % human platelet lysate (HPL) obtained from platelet-rich plasma procured by our Province of Buenos Aires Blood Bank. Few MSCs cells were present in the initial inoculum, but after culture, they expanded rapidly exponentially. MSCs appeared soon as typical round colonies, and a monolayer started to form over 14–17 days. When an 80 % of confluence was obtained, MSCs were detached from the culture flasks, expanded in a second passage, phenotyped as CD105-, CD73-, CD44-, and CD90-positive cells, lacking CD45, CD34, CD14, CD11b, CD79, CD19, and HLA-DR surface molecules expression, microbiologically tested, karyotyped as normal, and cryopreserved as needed. Cultured cells were daily examined under a phase contrast inverted microscope. The final samples before implantation were always negative as tested for adventitious agents, and small amounts of all of them were kept in quarantine for a period of time after transplantation. There is not clearly a certain or definitive amount of cells yet to be used in these procedures, and the selected concentration for this trial was based in previous work done by us in animal studies and data presented by others before in different reports. A final suspension of MSCs at a concentration of 1 × 10^6^ cells/ml was prepared from the adherent cells in the plastic flasks and mixed in a fraction of cryoprecipitate from donor blood, with high amounts of fibrinogen and other clotting factors, obtained from the same blood bank as the HPL. A second component was also prepared with thrombin (Tissucol, Baxter) at a concentration of 5 U/ml of PBS.

### Repairment of burn wound with CMSCs

**Fig. 1:**
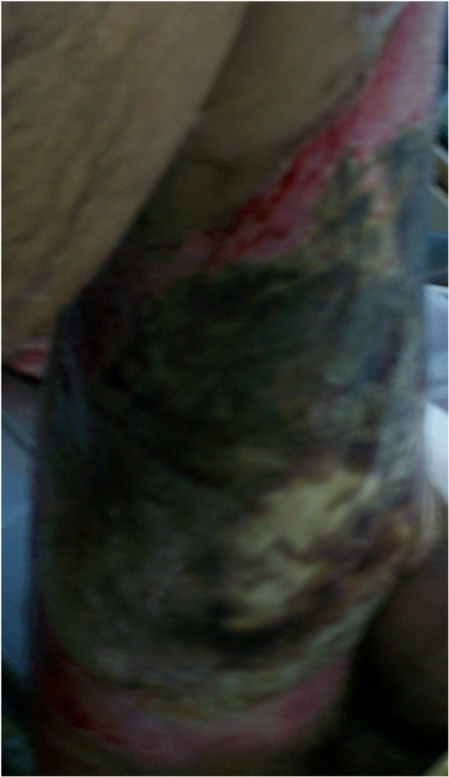
Deep burn on the right thigh. Necrotic burned tissue was observed in the right thigh of the patient at admission to the hospital

**Fig. 2:**
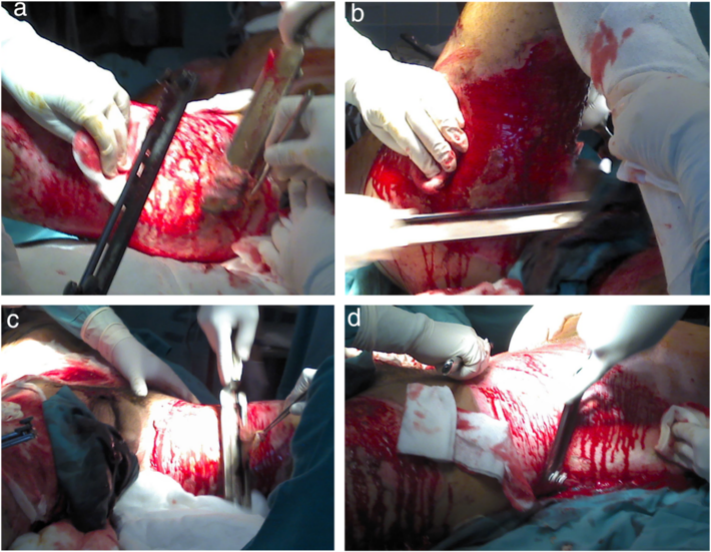
Deep early escharotomy. **a**–**d** Escharotomy of severely burned tissue was performed during the first surgery

A 26-year-old, young healthy male patient was admitted to the Burns Unit at the San Martín Hospital, Province of Buenos Aires Ministry of Health, in La Plata, Argentina, on November 3, 2011, presenting with severe thermal (flame) burns of both lateral and anterior parts of the abdomen, left and right armpits and tights, parts of the buttocks and the back, and total area up to 60 % body surface (30 % full-thickness burn) (Fig. 1). He told at the emergency room that his clothes inflamed during fire while using a grass trimmer that exploded. The burned surfaces were washed, and supportive therapy was administered to the patient. As the patient’s clinical condition improved, combined treatment by traditional and new methods was started, including early deep escharotomy of necrotic tissue (Fig. 2), cell therapy with CMSCs, and posterior autologous meshed skin grafting (AMSG) if needed.

Both solutions as mentioned in the “Obtention and preparation of human cadaveric MSCs for transplantation” section, one with the fibrinogen and MSCs and the other one with thrombin, were introduced and preserved in two different sterile conical tubes at room temperature, transported within the next hour to the Burns Unit at the San Martín Hospital of the Province of Buenos Aires, Ministry of Health in La Plata, Argentina, and sprayed onto the burn wound surface of the patient after deep escharotomy at a density of 1 × 10^6^ MSCs per 100 cm^2^, using a “duplo-jet” system. In this way, thrombin polymerized the fibrinogen of the cryoprecipitate fraction and adhered the cells within a friendly fibrin matrix to the injured surface immediately as it was sprayed over the wounded area. After cell transplantation, the burned surfaces were dressed with a sterile polymeric transparent film that was first changed on day 7 and every other 2/3 days later on. A total of two main courses of this kind of cell therapy were administered to the wounded areas with 2-week difference between each other. Wound healing process and efficacy of the cell therapy was evaluated visually during wound observation on days 1, 3, 6, 7, 9, 11, and 13 and then as needed. As we focused on the question of stem cell persistence and its detection in the treated wound bed after its implantation, we decided for practical methodology and ethical reasons not to do so in this moment besides that it could have been a great idea to finally elucidate MSC fate and possible differentiation times and pathways when they have been transplanted in a human being from an allogeneic source as our cadaver donor. Also, as there is no evidence of immunological rejection events by using allogeneic MSCs, we have designed our procedure using CMSCs without the need of HLA matching between the donor and recipient. Anyway, we performed HLA typification of both the donor and the patient’s cells. Also, we looked for microchimerism [14] as well as any possible adverse effect related to immune intolerance or rejection. It is very important to emphasize that we were ready to document any possible adverse effect at any time, with safety as being one of the main end points of the trial. We have used as control group all patients with similar type of skin lesions treated in our Burn Unit with standard therapies in the past 10 years. Our efficacy end points were mortality rate, days to complete healing of the lesions, hospitalized total days, amount of surgery procedures performed, functional sequelae, quality of the regenerated skin, and infectious complications, among many others. As this is only one patient presented here, a statistical analysis was not carried on yet to see the differences in wound healing between CMSC-treated wounds and non-CMSC-treated wounds.

## Results

**Fig. 3:**
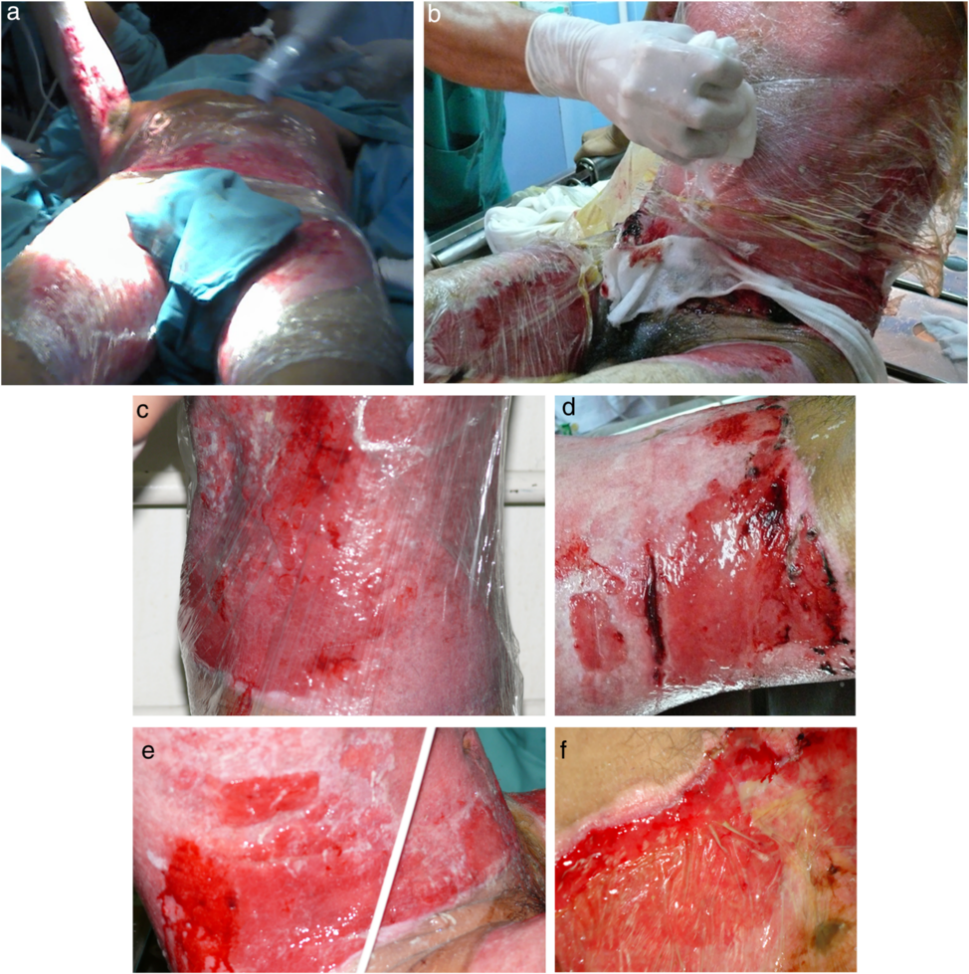
Burn wounds were treated with cadaveric bone marrow mesenchymal stem cells (CMSCs) and covered by a transparent polymeric film. **a** The patient at 1 week after deep escharotomy and first treatment with CMSCs and films. All the lesions have been washed and new film applied. Right axilla was also seen before film application. **b** First film change at first week after the CMSC treatment. The granulation-like tissue was seen under the film without much exudate. **c**–**e** One week after the first CMSC treatment, a granulation-like tissue was seen before first film replacement. The granulation-like tissue was probably a very early vascularized “neo-dermal like matrix” after CMSCs treatment under an active growth and differentiation natural program. This new matrix behaved as a novel and may be also “universal” scaffold allowing very well for autologous meshed skin graftings (AMSGs) attachment and maybe later appearance of keratinocytes and the production of a mature epidermis. **f** Wound epithelialization at second week after treatment with CMSCs. Rapid epithelialization from the edges and some epithelial growth in the center of the lesion was observed

**Fig. 4:**
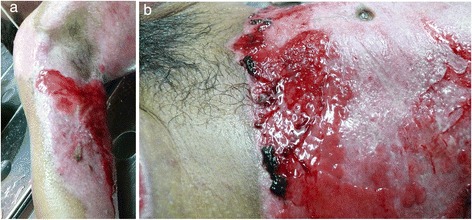
Wound appearance without film at third week after first treatment with CMSCs. **a** Right axilla with granulation-like tissue was seen without film. **b** “Incipient spontaneous” epithelialization in left flank. Hyper-proliferation of the granulation-like tissue was rapidly observed in all areas treated with CMSCs

**Fig. 5:**
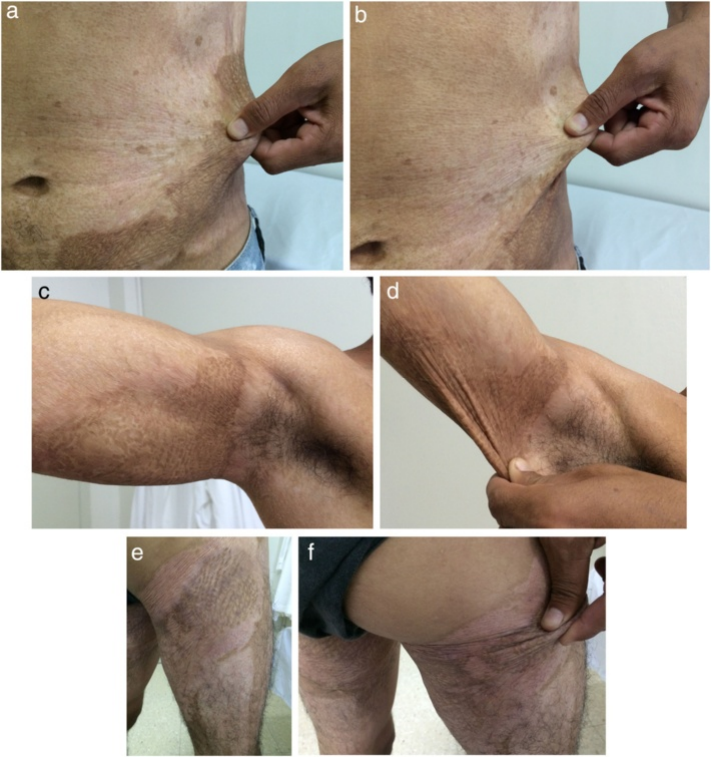
Burn wounds recovered at patient discharge. **(a**–**f)** At patient discharge, a slow integration of the grafts with progressive disappearance of their meshed aspect seems to be taking place, looking more like normal skin. Good elasticity of the whole skin, almost no functional sequelae, limited hair regrowth were observed in some areas in which MSCs have been applied

**Fig. 6:**
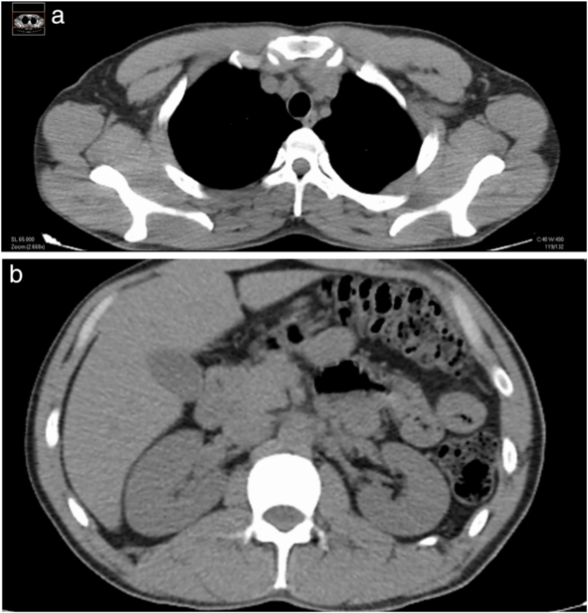
**a–b** CT scan studies. In CT scan studies, almost no skin retraction at any site is observed. Deep dermis as well as the epidermis seem to be normal again

Surgical complete debridement of the necrotic burned tissues were performed soon after admission of the patient and cell therapy applied immediately (Fig. 2). Probably due to some potential yet unknown properties of MSCs or any of its transportation materials, or by the simultaneous use of the polymeric transparent film as a dressing, lost of plasma was not a concern, necrotic tissues were not ever seen again after escharotomy at any site, and good blood supply without wound infection was always present after application of the stem cells (Fig. 3a). Bacteriological analysis of the wound surfaces and clinical observation did not show evidence of local infection at any time. Burn wounds were only washed with warm water and soap at the end of the first week after the cell treatment and every third or fourth day later on, always covering the lesions with the polymeric film. Antiseptics were not used at any time. The transparent film dressing did not ever stick to the wounded areas, and a yellowish fibrinoid material was always present and seen under it (Fig. 3f). Besides this observation, other exudates were minimun under our film not needing any special drainage. An increasing progressive development of new capillaries were soon observed in the surface of all treated wounds. A “granulation tissue-like” appearance was evident in all the wounds by the fifth day, and from this moment, “hyper-granulation” also developed and persisted on all treated burn wounds (Fig. 3a–f). There was a fast and significant improvement of the clinical condition of the patient, and pain in the burned areas decreased in intensity. A surprisingly early and not very well adherent thin epithelial growth was seen rapidly advancing from the wound edges in relation with the topical cell therapy, but the “granulation-like” wound surface remained almost unchanged in the center (Fig. 4a–b). After 35 days of treatment with two courses of CMSCs application as described, the complete epithelialization of the wounds seemed to be too slow to be waited further. Then, after superficial surgical shaving of all the “hyper-granulating” areas, AMSG was applied. Skin grafts were obtained from the non-burned zones, meshed and used to cover the wound surfaces on every site. About 50 % of the remaining wound surface was covered during a first operation, and additional transplantation of more auto-grafts were done after another week always using the polymeric film as a dressing. All AMSGs permanently adhered to the wounded surfaces and were always viable. Interestingly, the spaces inside the meshes seemed to rapidly and completely epithelialized in a very short period of time. The patient keeps continuously improving day after day, leaving the bed and starting walking and moving with significant freedom. He was finally discharged from the hospital in a very well satisfactory condition for outpatient rehabilitation and follow-up (Fig. 5a–f). More interesting is the fact that in the sites in which AMSGs were applied, a slowly progressive integration of the grafts with disappearance of their meshed aspect seems to be taking place, looking more like normal skin. Almost no retraction is seen at any site, and the deep dermis as well as the epidermis seem to be normal again as observed in CT scan studies (Fig. 6a–b).

## Discussion

### Similarity of results obtained in this study and by Rasulov’s group

Our results seem to be very similar to those described by the Rasulov’s group [1]. Both, Rasulov and us, have seen an interesting fasting growth not only of a “granulating dermal-like” tissue but also of a limited new epidermis mainly from the wound edges. At the same time, local infection or necrosis do not seem to be big concerns while using this type of therapy. There was a rapid development of neo-angiogenesis in the lesions treated with MSCs with a simultaneous growth of a granulation-like tissue. Surprisingly, all these surfaces never showed any sign of infection or necrosis. Incipient epithelialization was observed by us soon after the first week of CMSC implantation, may be induced by the capacity of these allogeneic CMSCs to promote epithelial and endothelial growth mainly as it was said before at the periphery of the wounds, decreasing the total burned area. Also, healing by fast epithelial growth was observed inside the meshed spaces of the AMSGs. In further evaluations of the treated deep wounds, limited hair regrowth was observed in some areas. We have seen and described similar results in our burned pig model [4]. Rasulov’s and our patient needed a final epithelium replacement as it was described. We used AMSGs while the other group applied “SGs” without mentioning if they were meshed or not. The accelerated growth of a new epithelium between the meshes that covered the entire empty spaces in a short period of time was really amazing in our patient.

### Differences observed between the study of Rasulov’s and our’s

Differences between this study and Rasulov’s [1] were observed by us: (1) the type of cells used by Rasulov were designated as “allogeneic fibroblast FMSCs” but their phenotype, features of the donor, testing for infectious agents, or other data of these cells were not described in their paper. Our’s were bone marrow MSCs obtained from a unique cadaver donor and fulfilled the phenotyping criteria for MSCs as well as all other requirements by local law to be transplantable including all the tests done to detect possible adventitious agents. No microchimerism was observed after several determinations done at different moments of the patient’s follow-up and no immune adverse events were seen at any time. (2) The Russian group did not specify by which way they applied the cells on the surface of the patient’s wounds. We sprayed the CMSCs into a blood cryoprecipitate that polymerized in situ. (3) Our cell culture method did not involve the use of bovine fetal serum but HPL. It was obtained from platelet-rich plasma having an outstanding potential to promote the growth of CMSCs while maintaining their phenotype and differentiation capacities. (4) We have also always used a transparent polymeric film to cover the wounds after the cell treatments. Rasulov’s group employed just gauze. It is interesting to say that every time stem cells with fibrin were used, a yellowish creamy material under the film was always observed with its characteristic appearance but without wound infection. This could be a promoting interesting material for the healing processes of the wounds that surely deserve further studies and maybe also the essence for a future kind of regenerative product. (5) The development of the granulation-like tissue and its “hyper-proliferation” was rapidly observed in all areas treated with CMSCs both by us (as we also did in our previous pig burned model) and Rasulov’s group.

### The granulation-like tissue

As far as we know, this is probably a very early vascularized “neo-dermal-like matrix” under an active growth and differentiation natural program, and not just simply a “granulation tissue”. This new matrix behaves as a novel and may be also as a “universal” scaffold allowing very well for AMSG attachment and may be later appearance of keratinocytes and the production of a mature epidermis. In this way, keratinocytes could come at an early time from the edges of the wounds or may be by direct differentiation from resident MSCs later on after neo-matrix organization [4].

### The first experience using CMSCs in the treatment of a burned patient

In any case, our future challenge in relation to the regeneration processes and in order to obtain a faster healing of severe deep burns as well as many other lesions or diseases of the body will surely be, as it was considered in our previous editorial to Burns [5], a more precise management and understanding of the variable of time. In this way, the needed length of time to completely regenerate a full-thickness wound by the simple application of CMSCs could be too long for this type of patients, even for the potential growth and differentiation capacities of these cells. In this scenario, we waited 35 days to apply the AMSGs, while Rasulov’s group did it as soon as 4 days after the cells were delivered on the wounds. Probably, it does not really mean that MSCs do not have the potential to regenerate a complete skin including the epidermis, only that maybe, more time could be needed as they have in this kind of deep burns a long harder complicated work to do by first rebuilding the vasculature and the matrix of a neo-dermis before they could take care of the final regeneration process for the development of a neo-epidermis. By now, these preliminary results are very promising by themselves. If time finally ends to be the critical point, we might have several new major options to be employed besides waiting for whole recovery by the simple action of the cells: (1) To early apply the AMSGs as soon as the granulation-like tissue induced by MSCs is seen as Rasulov did. (2) To put together the MSCs and the AMSGs or one specially made with “very wide meshes”, expecting that the growth promotion advantages given by MSCs would be stimuli which is enough to rapidly regenerate the whole skin in a shorter period of time while needing and using much less residual healthy skin from the patients for the auto-grafting. (3) To early use cultured keratinocytes/epidermal grafts or just keratinocyte stem cells with MSCs. (4) To deliver MSCs in novel different ways like in the shape of “spheroids” as we have previously described [15]. This could accelerate the time to complete regeneration specially if keratinocytes could also be grown in the outer surface of these spheroids. (5) To potentially use “intelligent matrices” with nanoparticles and stem cells to accelerate the time for healing [4]. It is important to say that the patient did not have any adverse effect at any time even after more than 3 years of follow-up. He always showed a very well tolerance to the treatment with CMSCs. At this moment, we see almost no scar or deformity in the places treated with CMSCs. The patient looks and feels clinically very good. There are no functional sequelae, and he re-started to work very soon after being discharged from the hospital.

## Conclusions

This is mainly a descriptive as well as an observational study of all the chronological events seen in this only one and “first patient” treated in the world with CMSCs. Our preliminary experience and our early results show an outstanding safety data in the use of CMSCs in our first severe burned patient treated in this way. Based on all this, we think that improvements in the use of CMSCs for burns might be possible in the near future, and a lot of time as well as many lives could be saved by many other research teams all over the world.
